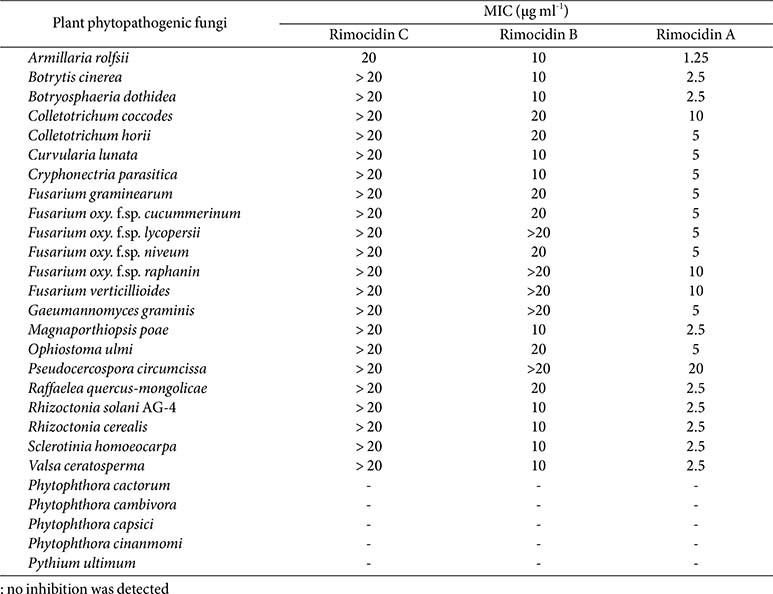# Harnessing Rimocidins-Producing *Streptomyces* sp. JCK-6116 as a Sustainable Fungicide for Biocontrol of Cucumber Soil-Borne Diseases

**DOI:** 10.4014/jmb.2508.08023

**Published:** 2025-10-28

**Authors:** Hang T. T. Nguyen, Loan Thi Thanh Nguyen, Ae Ran Park, Van Thi Nguyen, Quang Le Dang, Jin-Cheol Kim

**Affiliations:** 1 Faculty of Applied Sciences, Ton Duc Thang University, Ho Chi Minh City, Vietnam; 2 Plant Healthcare Research Institute, JAN153 Biotech Incorporated, Gwangju 61186, Republic of Korea; 3 Department of Agricultural Chemistry, Institute of Environmentally Friendly Agriculture, College of Agriculture and Life Science, Chonnam National University, Gwangju 61186, Republic of Korea; 4 Institute of Materials Science, Vietnam Academy of Science and Technology, Hanoi 10072, Vietnam; 5 Graduate University of Science and Technology, Vietnam Academy of Science and Technology, Hanoi 10072, Vietnam

**Keywords:** Antifungal activity, rimocidins, *Streptomyces*, fusarium wilt, damping-off disease, biocontrol

## Abstract

*Fusarium oxysporum* f. sp. *cucumerinum*, the causal agent of cucumber fusarium wilt, along with *Rhizoctonia solani* AG-4 and *Pythium ultimum*—that causes cucumber damping-off—are soil-borne fungal and Oomycetes pathogens responsible for significant economic losses in agriculture. In this study, the culture filtrate of *Streptomyces* sp. JCK-6116, isolated from soil, exhibited strong inhibitory activity against the mycelial growth of multiple phytopathogenic fungi in a 96-well microtiter plate assay. In the dual culture assay, JCK-6116 inhibited the growth of 20 species of plant pathogenic fungi and Oomycetes, suggesting a wide antifungal spectrum. Three active compounds—rimocidin A, B, and C—were isolated from JCK-6116 and identified. These rimocidins exhibited antifungal effects against fungi by binding to ergosterol in the fungal membrane. However, none of the compounds exhibited anti-oomycete activity against the tested Oomycetes strains. Among the three compounds, rimocidin A demonstrated the strongest antifungal activity with minimum inhibitory concentration values ranging from 1.25–10 μg/ml. Furthermore, the culture broth of JCK-6116, at 10-fold dilution, effectively suppressed fusarium wilt and the two damping-off diseases in cucumber. Its butanol extract was also effective against the two fungal diseases but showed no activity against *P. ultimum* damping-off disease. These findings indicate that the culture broth contains metabolites with anti-oomycete activity. This study demonstrates that *Streptomyces* sp. JCK-6116 has significant potential as a biological control agent for managing soil-borne diseases caused by fungi and Oomycetes.

## Introduction

Cucumber (*Cucumis sativus* L.) is a globally significant crop with widespread cultivation. Its nutritional value is notable for a high content of vitamins (K, C, and B1), minerals, and water [1]. The expansion of cultivation in recent years has shifted production toward a large-scale model [2]. However, cucumbers remain vulnerable to a wide range of diseases throughout their development.

Fungal and Oomycetes pathogens, particularly *Fusarium oxysporum*, *Rhizoctonia solani*, and *Pythium ultimum*, cause significant losses in cucumber production. Fusarium wilt can affect cucumber plants at any developmental stage, with yield reduction ranging from 15–50%, owing to reduced phytosynthesis [3]. In some cases, total crop loss may occur. Damping-off—primarily caused by *R. solani* and *P. ultimum*—is one of the most prevalent and destructive diseases affecting cucumber seedlings [4, 5]. *Pythium* spores germinate and directly penetrate feeder roots [5], while sclerotia of *R. solani* infect young plant tissues [4], thereby disrupting normal plant development. The management of phytopathogenic diseases currently relies predominantly on chemical fungicides. However, the repeated application of chemical fungicides has led to several adverse consequences, including accumulating chemical residues and the emergence of resistant pathogen strains. Over the past few decades, numerous instances of fungicide resistance have been documented globally. For example, *Erysiphe graminis* developed resistance to strobilurins (quinone outside inhibitors), which are used to control powdery mildew on cereals, within 2 years of application [6]. These challenges have motivated many researchers to explore effective alternative strategies for managing soil-borne fungal diseases. The use of biocontrol agents has emerged as a promising and sustainable alternative strategy for managing plant diseases in recent decades.

Microorganisms are widely recognized as effective alternative strategies for controlling phytopathogenic fungi. Several genera—including *Bacillus*, *Streptomyces*, *Pseudomonas*, and *Paenibacillus*—have been identified as biocontrol agents owing to their ability to produce various antifungal metabolites [7-9]. Among these, *Streptomyces* species have significant potential for producing agricultural valuable antifungal compounds [10]. These bacteria are abundant and widely distributed in soil, constituting approximately 40% of the soil bacterial community, particularly in the rhizosphere, and can also be found in water, air, plants, and other environments [11, 12]. Unlike Gram-negative bacteria, *Streptomyces* species are rarely pathogenic to plants, animals, or humans. This genus is responsible for producing approximately 60% of the antibiotics used in agriculture [13]. *Streptomyces* species can promote plant growth by producing phytohormones, siderophores, nitrogen fixation, enhanced nutrient bioavailability, and alleviating abiotic stress. Furthermore, several *Streptomyces* species can control pests, plant diseases, and weeds by producing various secondary metabolites, including hydrolytic enzymes and antibiotics [14]. Numerous naturally occurring fungicides—including polyenes, kasugamycin, validamycin, blasticidin S, and polyoxin—have been isolated from *Streptomyces* spp. [15, 16].

Polyenes are broad-spectrum antifungal metabolites characterized by a cyclic amphiphilic macrolide substructure and are primarily derived from *Streptomyces* species [17]. This group typically features a polyketide core macrolactone ring with 20–40 carbon atoms, including 3–8 conjugated double bonds [18]. Commercially important polyenes include amphothericin B, nystatin, natamycin, and candicidin. Previous studies show that polyenes exert their antifungal activity through several mechanisms of action. These mechanisms include increasing membrane permeability, inducing the production of reactive oxygen species, and forming sterol sponges by extracting ergosterol from the fungal membranes [19-22]. Rimocidin—a member of the polyene group—was first isolated and identified from *S. rimocus* [23]. Rimocidin A and B (BU16) at 1 μg/ml significantly reduced anthracnose symptoms caused by *Colletotrichum coccodes* compared to that of chlorothalonil applied at 50 μg/ml after 2 days of inoculation [24]. Rimocidin C (also known as CE-108)—isolated from *Streptomyces diastaticus* 108 [25]—exhibits antifungal activity that is reported to be lower than that of rimocidin A. Although rimocidin A, B, and C have been identified and their biological activities characterized, no study has reported the simultaneous production of all three rimocidins by a single *Streptomyces* strain and directly compared the antifungal activities of these three compounds.

*Streptomyces* sp. JCK-6116 demonstrates strong *in vitro* antifungal activity against several fungal pathogens following a screening of 120 actinomycete isolates obtained from soil. Therefore, this study aims to (1) isolate and identify JCK-6116; (2) purify and characterize the antifungal metabolites produced by this strain; (3) assess the antifungal activities of rimocidin and its two derivatives isolated from *Streptomyces* sp. JCK-6116; and (4) evaluate the disease control efficacy of the fermentation broth and the crude extract of JCK-6116 against fusarium wilt and damping-off in cucumber plants.

## Materials and Methods

### Culture and Identification of JCK-6116

Strain JCK-6116 was isolated from soil collected in South Korea and maintained on Bennet’s agar medium, consisting of beef extract (1 g), glucose (10 g), enzymatic digest of casein (2 g), yeast extract (1 g), agar (15 g), and distilled water (1 L).

JCK-6116 was cultured on International *Streptomyces* Project (ISP) media 1 through 5, Bennet’s agar, and potato dextrose agar (PDA) to observe the color of aerial mycelium, substrate mycelium, and soluble pigments. Carbon utilization and enzyme activity of JCK-6116 were evaluated using API 50 CH and API ZYM systems (bioMérieux, France). Cellulase, protease, and chitinase activities of JCK-6116 were determined by culturing this strain on media containing the respective substrates.

The taxonomic position of strain JCK-6116 was determined through 16S rRNA sequencing. The 16S rRNA gene was amplified and sequenced using the universal primer set 27F/1492R [26]. Phylogenetic relationships were inferred using the neighbor-joining method [27]. The optimal phylogenetic tree is presented with branch lengths proportional to evolutionary distances [28], measured in substitutions per site. Bootstrap support values, based on 1,000 replicates, are indicated at the corresponding branches and represent the percentage of replicate trees in which the associated taxa clustered together [29]. Evolutionary distances were calculated using the Kimura 2-parameter method [28] and were expressed as the number of base substitutions per site. Phylogenetic analyses were conducted using MEGA X software [30].

### Plant Pathogenic Fungi

Twenty-six true fungi and Oomycete species were used in this study, including *Armillaria rolfsii* (*Ar*), *Botrytis cinerea* (*Bc*), *Botryosphaeria dothidea* (*Bd*), *Colletotrichum coccodes* [14], *C. horii* (*Ch*), *Curvularia lunata* [19], *Cryphonectria parasitica* (*Cp*), *Fusarium fujikuroi* (*Ff*), *F. graminearum* (*Fg*), *F. oxysporum* f.sp. *lycopersii* (*Fol*), *F. o.* f.sp. *niveum* (*Fon*), *F. o.* f. sp. *raphanin* [31], *F.o.f.* sp. *cucumerinum* (*Foc*), *F. verticillioides* (*Fv*), *Gaeumannomyces graminis* (*Gg*), *Magnaporthiopsis poae* (*Mp*), *Ophiostoma ulmi* (*Ou*), *Pseudocercospora circumciss*, *Raffaelea quercus-mongolicae* (*Rm*), *Rhizoctonia solani* (*Rs*), *R. cerealis* (*Rc*), *Clarireedia jacksonii* (*Clj*), *Valsa ceratosperma* (*Vc*), *Phytophthora cactorum* (*Pcac*), *Phy. cambivora* (*Pcam*), *Phy. capsici* (*Pcap*), *Phy. cinanmomi* (*Pcin*), and *Pythium ultimum* (*Pu*). The fungal species names, their origins, and associated plant diseases are listed in Table S1. Growth conditions for the pathogenic fungi were described in our previous report [32].

### Dual Culture Assay

The antagonistic activity of JCK-6116 against several plant pathogenic fungi was evaluated using a dual culture assay on PDA, following a previous report [33] with slight modifications. A 5-mm diameter agar plug of each target fungus was placed 2 cm from the edge of the plate, while JCK-6116 was streaked onto the plate 4 cm away from the fungal plug. Control plates were inoculated with the fungal pathogen alone. All plates were incubated at 25°C for 5–14 days until the mycelial growth of the control fungus completely covered the plate. The inhibition rate of JCK-6116 against pathogenic fungi was calculated using the following formula:

$$\text{Inhibition rate =}\frac{\left(a-b\right)}{a}\times 100\%$$

Where a represents the radial mycelial growth of the fungus on the control plate, and b represents the radial mycelial growth of the fungus on the dual culture plate.

### Extraction and Isolation of Active Metabolites

To produce antifungal metabolites, *Streptomyces* sp. JCK-6116 was incubated in GSS medium at 28°C with shaking at 180 rpm for 7 days. The resulting 2.5 L culture filtrate of JCK-6116 was extracted twice with butanol (BuOH), and the organic phase was concentrated using a rotary vacuum evaporator (N-1110, EYELA Co., Japan), yielding 6.25 g of crude extract. The crude extract was suspended in distilled water at pH 10 to remove insoluble impurities. Subsequently, the pH was lowered to 7 to precipitate insoluble metabolites. The rimocidin mixture was then purified from the precipitate by preparative high-performance liquid chromatography (HPLC) using an XBridge OBD Prep C18 column (5 μm, 19 × 250 mm) (Waters, USA). The mobile phase comprised eluent A and B (water and MeOH, respectively). Elution was performed at a flow rate of 15 ml/min using a linear gradient of 40%–75% B over 30 min. Active compounds were detected at 303 nm. Target fractions were collected and evaporated to dryness, yielding compounds **1** (26.6 mg), **2** (9.7 mg) and **3** (5.4 mg).

### Structural Identification of Isolated Active Metabolites

The purified compounds were characterized by ^1^H-NMR, ^13^C-NMR, ^1^H – ^1^H COSY, ^1^H – ^13^C HMBC, and (^1^H, ^13^C) HSQC spectroscopy using a Bruker Avance III HD 500 MHz spectrometer (Bruker Biospin GmbH, Germany). DMSO-*d*_6_ (Cambridge Isotope Laboratories, Inc., USA) served as the solvent, with tetramethylsilane as the internal standard. Molecular weights were determined by electrospray ionization mass spectrometry (ESI-MS), operating in negative and positive modes using a UPLC Q-TOF mass spectrometer (Waters Corp., UK) equipped with a photodiode array detector [34].

**1:** pale yellow solid; ^1^H and ^13^C NMR data, see Table 1; HRESIMS *m/z*: 766.4040 [M − H]^−^, and *m/z*: 750.4076 [M + H – H_2_O]^+^ (calcd for C_39_H_60_NO_14_, 766.8839, and for C_39_H_60_NO_13_, 750.8845)

**2:** pale yellow solid; ^1^H and ^13^C NMR data, see Table 1; HRESIMS *m/z*: 752.3870 [M − H]^−^, and *m/z*: 736.3914 [M + H – H_2_O]^+^ (calcd for C_38_H_58_NO_14_, 752.8576, and for C_38_H_58_NO_13_, 736.8582)

**3:** pale yellow solid; ^1^H and ^13^C NMR data, see Table 1; HRESIMS *m/z*: 738.3721 [M − H]^−^, and *m/z*: 722.3774 [M + H – H_2_O]^+^ (calcd for C_37_H_56_NO_14_, 738.8313, and for C_37_H_56_NO_13_, 722.8319)

### Determination of the Content of Rimocidin A in the Culture Filtrate and Crude Extract

Isolated rimocidins A, B and C (> 95% purity) was used as the reference standard to quantify three rimocidins in the culture filtrate and crude extract of JCK-6116. Eight solutions of rimocidins were prepared in DMSO at concentrations of 0.46, 1.37, 4.12, 12.35, 37.04, 111.11, 333.33, and 1,000 μg/ml. A 1 μl aliquot of each rimocidins standard, JCK-6116 culture filtrate, and crude extract were injected into the HPLC system (Shimadzu Scientific Instruments, Japan). Peak areas corresponding to rimocidins were measured for each sample. A standard curve was constructed by plotting the peak areas of the purified rimocidins standards. Using this curve, the concentrations of rimocidins in the culture filtrate and crude extract were determined. All quantitative analyses were conducted in triplicate.

### Evaluation of *in vitro* Antifungal Activities of Rimocidins

The antifungal activity of the three rimocidins was evaluated using the broth dilution method in 96-well microplates [34]. Stock solutions of each metabolite were prepared at 2 mg/ml in DMSO and subsequently tested across a concentration range of 0.625–20 μg/ml. A 1% DMSO solution was used as the untreated control. Plates were incubated at 25°C for 2–7 days, depending on the pathogen. The minimum inhibitory concentration (MIC) was defined as the lowest concentration that completely inhibited mycelial growth.

### *In planta* Bioassays

To evaluate the biocontrol efficacy of JCK-6116 against cucumber diseases, the Chungboksamchok and Nebakja cucumber varieties (FarmHannong, Republic of Korea) were selected for assessing fusarium wilt and damping-off, respectively. Germinated seeds were planted in nursery soil and maintained under 25°C with 75%relative humidity under a 16:8-h light/dark photoperiod. The JCK-6116 culture broth was diluted 10- and 100-fold with distilled water prior to application. Additionally, the BuOH extract was dissolved in DMSO at 30 mg/ml and further diluted with distilled water to final concentrations of 300 and 50 μg/ml. For positive controls, the synthetic fungicides Kajiran (containing 10% etridiazole and 55% thiophanate-methyl; Farmhannong Co., Ltd., Republic of Korea) at 1 g/l, and Jalrokend (containing 30% hymexalzol and 5% penthiopyrad; HankookSamGong Co., Ltd., Republic of Korea) at a 1,000-fold dilution were used to manage fusarium wilt and damping-off, respectively. All samples were supplemented with Tween 20 at a concentration of 250 μg/ml. Glucose Starch Soybean meal medium containing Tween 20 served as the untreated control.

### Disease Control Efficacy of JCK-6116 Fermentation Broth and Crude Extract against Cucumber Fusarium wilt Caused by *Fusarium oxysporum* f. sp. *cucumerinum*

Seven-day-old cucumber seedlings were treated with 20 ml of each sample by soil drenching 24 h before inoculation with 20 ml of microspore suspension (10^6^ spore/ml) of *Foc*. Disease incidence was assessed after 4 weeks of growth. Disease severity was calculated using the following scale [26]: 0, no symptoms; 1, yellowing and/or necrosis on < 25% of the leaf area; 2, yellowing and/or necrosis covering 26%–50% of the leaf area; 3, yellowing and/or necrosis covering 51%–75% of the leaf area; and 4, wilting, yellowing, and/or necrosis covering 76%–100%of the leaf area. The experiment was conducted in triplicate, with six plants per treatment.

### Disease Control Efficacy of JCK-6116 Fermentation Broth and Crude Extract against Cucumber Damping off Caused by *Rhizoctonia solani*

The biocontrol potential of JCK-6116 against *Rhizoctonia solani*-induced post-emergence damping-off was evaluated by soil drenching on 7-day-old cucumber seedlings, following the previously described method [35]. The disease severity of each plant was assessed after incubation at 25°C for 3–5 days.

### Disease Control Efficacy of JCK-6116 Fermentation Broth and Crude Extract against Cucumber Damping off Caused by *Pythium ultimum*

Seven-day-old cucumber seedlings were transferred to pots containing nursery soil inoculated with *P. ultimum* sporangia at a concentration of 10^3^/g soil [36]. Three days after transplantation, the disease incidence was assessed by counting the number of healthy cucumber seedlings. The experiment was conducted in triplicate and repeated three times. The mean of six replicates per treatment was calculated and expressed as a percentage relative to the control.

### Statistical Analysis

Data from the dual culture assay and evaluating the efficacy of JCK-6116 against phytopathogenic fungal diseases are presented as means ± standard deviation (SD) of the replicates. Experimental results were analyzed using one-way ANOVA followed by Duncan's multiple range test (SPSS version 26.0, USA). Statistical significance was set at *p* < 0.05.

## Results

### Characterization of Strain JCK-6116

JCK-6116 exhibited robust growth across all tested media and produced a brown, water-soluble pigment on ISP1 and ISP2 media. JCK-6116 produced distinct coloration of aerial and substrate mycelia depending on the growth media. White aerial mycelium was observed on ISP1, ISP2, ISP5, Bennet’s, and PDA media, whereas it appeared white-violet and white-pink on ISP3 and ISP4, respectively. Substrate mycelium coloration varied and included shades of white, pale yellow, grey, brown, and dark brown (Table S2). Furthermore, JCK-6116 utilized only two carbohydrate sources—esculin and D-sucrose—and produced several enzymes such as protease and chitinase (Tables S3 and S4), but its carbon utilization profile differed in some aspects from that of *S. mauvecolor* ATCC 29835 (Table S5).

### Identification of JCK-6116

**Fig. 1:**
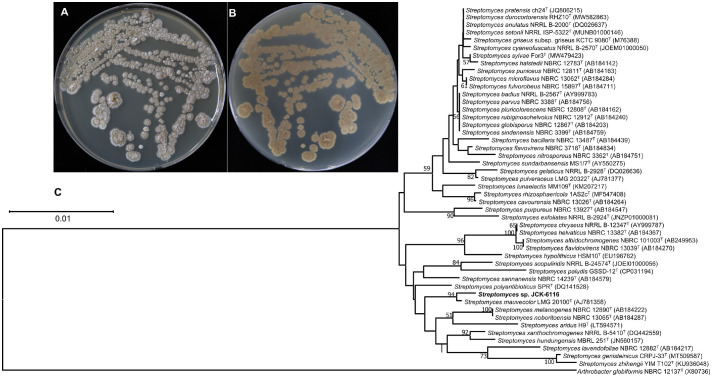
Growth and morphology of *Streptomyces* sp. JCK-6116 on ISP2 agar. (**A**) Top view and (**B**) bottom view of colonies grown on TSA plates for 7 days at 28°C; (**C**) Neighbor-joining phylogenetic tree based on 16S gene sequence of strain JCK-6116 and related *Streptomyces* species. *Arthrobacter globiformis* NBRC 12137^T^ was used as the outgroup. Bootstrap values (≥ 50%) from 1,000 replications are indicated at branch nodes. The scale bar indicates the number of nucleotide substitutions per site. TSA, tryptic soy agar.

Comparison of the 16S rRNA gene sequence of JCK-6116 (GenBank Accession No. PQ866075) with sequences in the GenBank database showed 100% similarity to that of *S. mauvecolor* 4219. Phylogenetic analysis using the neighbor-joining method confirmed that JCK-6116 clustered within a monophyletic clade alongside *S. mauvecolor* LMG20100 (Fig. 1). Therefore, these findings indicate that JCK-6116 belongs to the genus *Streptomyces*.

### Antagonistic Activity of JCK-6116 against Mycelial Growth of Phytopathogenic Fungi

**Fig. 2:**
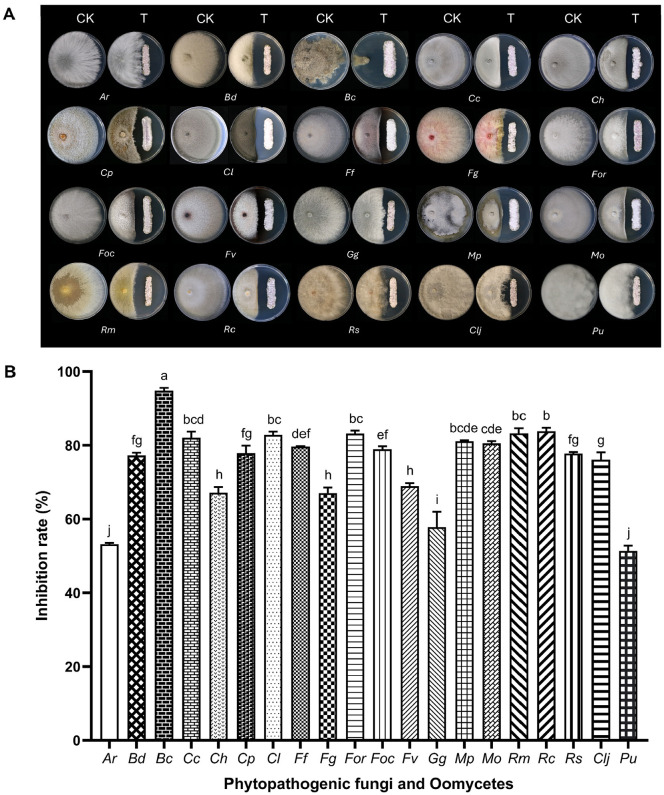
Antifungal activity of *Streptomyces* sp. JCK-6116 against 20 phytopathogenic fungi and Oomycetes on (A) dual culture assay and (B) percentage of pathogen growth inhibition.

JCK-6116 exhibited a broad-spectrum inhibitory activity against the mycelial growth of all tested phytopathogenic fungi after 5–14 days of incubation in a dual culture assay (Fig. 2). Among the pathogens tested, *Bc* showed the highest inhibited sensitivity, with a mycelial growth inhibition rate of 94.8%. Furthermore, JCK-6116 strongly inhibited the mycelial growth of *Bd*, *Cc*, *Cp*, *Cl*, *Ff*, *For*, *Foc*, *Mp*, *Mo*, *Rm*, *Rc*, *Rs*, and *Clj*, each exhibiting an inhibition rate > 70%. Moderate inhibition (> 50%) was observed against the other fungi, including *Ar*, *Ch*, *Fg*, *Gg*, and *Pu*, compared to that of the negative controls.

Moreover, the culture filtrate of JCK-6116 inhibited the mycelial growth of all tested fungi and Oomycetes, with an MIC value of 0.16–2.5% (Table S6). JCK-6116 exhibited the strongest inhibition against *R. solani* AG-4, the causal agent of damping-off disease in cucumber, with an MIC value of 0.16%. *S. homoeocarpa* and *R. solani* were also completely inhibited by this strain at MIC values of 0.63 and 0.31%, respectively (Table S6). These findings indicate that JCK-6116 has the potential to serve as a biological control agent for managing fungal plant diseases.

### Isolation and Identification of Antifungal Metabolites from *Streptomyces* sp. JCK-6116

**Fig. 3:**
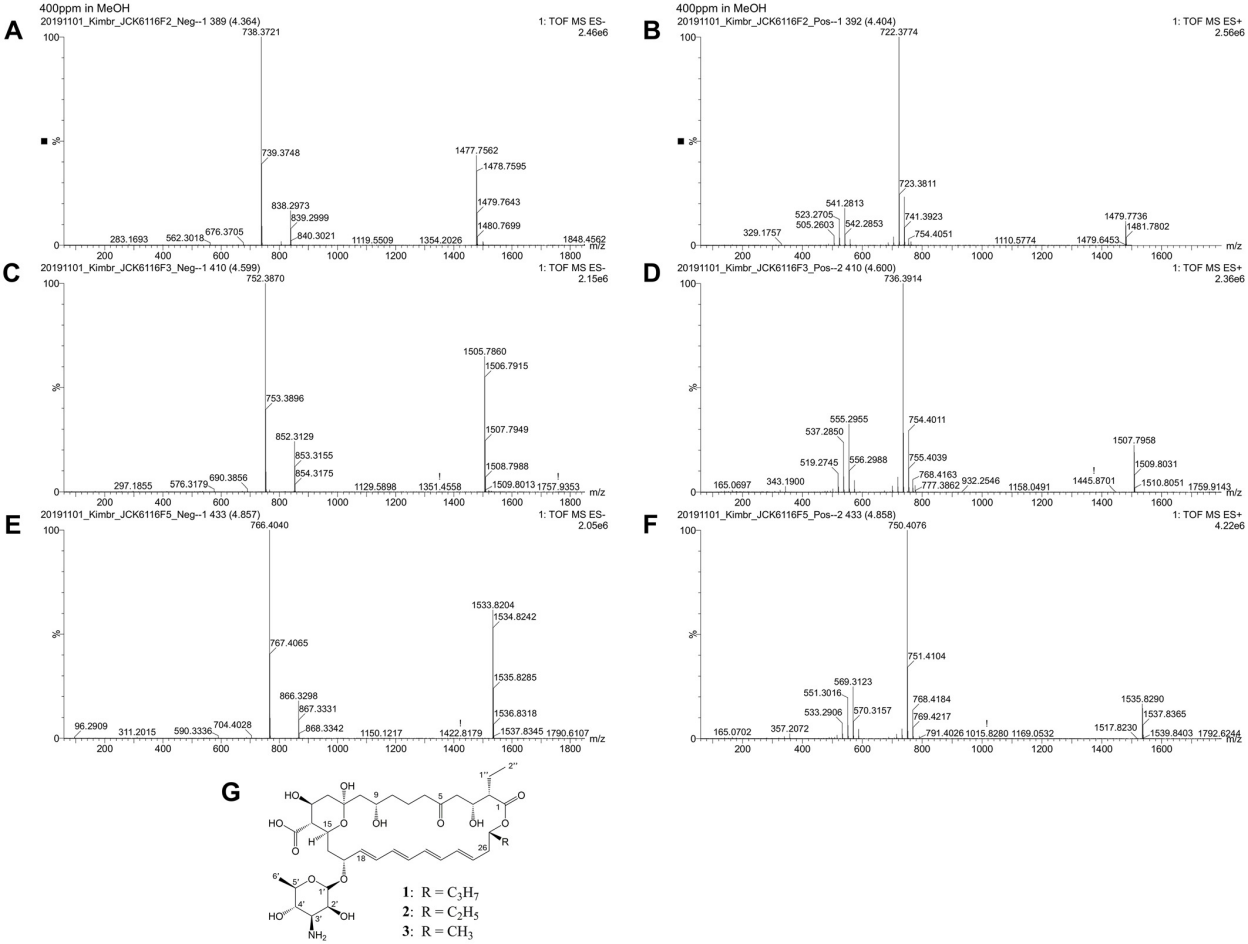
LC-ESI-TOF-MS spectra in negative and positive ion mode of rimocidin C (A-B), rimocidin B (C-D), rimocidin A (E-F) and chemical structure of rimocidins (G).

Three compounds (**1**, **2**, and **3**) were purified as yellow solids from the BuOH extract of *Streptomyces* sp. JCK- 6116. HPLC analysis revealed retention times of 19.6, 20.8, and 22.1 min for compounds **1**, **2**, and **3**, respectively. The UV spectra of these purified compounds exhibited absorption maxima at 291, 304, and 318 nm (Fig. S1). Molecular weights were determined for all three compounds using ESI-MS. Compound **1** exhibited negative and positive pseudomolecular ion peaks at *m/z* 766.4040 [M-H]^-^ and *m/z* 750.4076 [M + H – H_2_O]^+^, respectively (Fig. 3A and 3B); compound **2** showed peaks at *m/z* 752.3870 and *m/z* 736.3914 (Fig. 3C and 3D); while compound **3** exhibited peaks at *m/z* 738.3721 and *m/z* 722.3774 (Fig. 3E and 3F). These data suggested the molecular formulas of compounds **1**, **2**, and **3** as C_39_H_61_NO_14_, C_38_H_59_NO_14_, and C_37_H_57_NO_14_, respectively. The ^1^H and ^13^C NMR spectral data of three compound are summarized in Table 1. The nuclear magnetic resonance (NMR) data of the macrolactone ring exhibited characteristic signals such as a ketone resonance to δ 208.85– 209.32 ppm, a hemiketal signal at C-11 from 96.9–97.6 ppm, and a free carboxylic group signal range from 173.6– 177.9 ppm. Additionally, the lactone carbonyl corresponded to the signal from 172.2–172.6 ppm (Table 1). The tetraene system, spanning from C-18–C-25 in the macrolactone ring, was characterized by olefinic proton signals from 5.58–6.32 ppm in the ^1^H-NMR spectra and eight sp^2^ carbon signals ranging from 128.75–137.29 ppm in ^13^C-NMR spectra. In the mycosamine moiety, polyol protons appeared between 3.18 and 4.56 ppm; a methine proton at 2.82–2.88 ppm indicated a linkage to an NH_2_ group; and a methyl group at C-6' resonated between 1.16 and 1.17 ppm. A mycosamine acetal carbon was identified by carbon signals ranging from 95.7–96.4 ppm, correlated with the anomeric proton H-1' at δ 4.55–4.56 ppm (Table 1). The structure of mycosamine moiety in compounds **1** and **2** was further confirmed by HMBC cross-peaks. COSY and HMBC correlations were also instrumental in distinguishing signals from the aliphatic side chain (R) at C-27 in the structures of compounds **1** and **2**. All 1D and 2D NMR data of three compounds have been deposited at NP-MRD with acession numbers were NP0351554, NP0351555, and NP0351556. Based on comparison with previously reported data, compounds **1**, **2**, and **3** were identified as rimocidin A, B, and C (Fig. 3G).

### *In vitro* Antifungal Activity of Rimocidins

The *in vitro* antifungal activity of rimocidins A, B, and C were assessed against the mycelial growth of 26 phytopathogenic fungi and Oomycetes using the broth dilution method. All three compounds inhibited the growth of true fungi tested but showed no efficacy against Oomycetes (Table 2). Among them, rimodicin A exhibited the strongest antifungal activity, with MIC values ranging from 1.25–10 μg ml^-1^. Rimocidin B exhibited antifungal activity that was 2–4 times lower than that of rimocidin A. Among the three compounds, rimocidin C demonstrated the weakest antifungal effect. These findings suggest a correlation between the complexity of the side chain attached to the macrolide ring in the rimocidin structure and the extent of cellular membrane disruption.

### Disease Control Efficacy of JCK-6116 against Cucumber Diseases

**Fig. 4:**
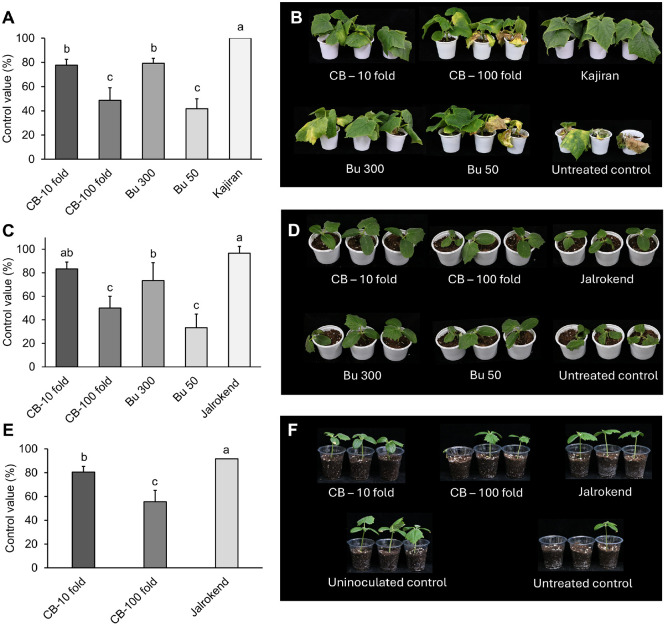
Disease control efficacy of culture broth and crude extract against plant diseases. (**A–B**) Fusarium wilt caused by *Fusarium oxysporum* f. sp. *cucummerinum*, (**C–D**) Damping-off caused by *Rhizoctonia solani*, (**E–F**) Damping-off caused by *Pythium ultimum*. CB: Culture broth tested at 10- and 100-fold dilutions. Bu: Butanol extract tested at 50 and 300 μg/ml. Commercial fungicides Kajiran and Jalrokend served as positive controls. Water-treated plants served as untreated controls.

The culture broth and BuOH extract of JCK-6116 significantly suppressed cucumber disease development in a dose-dependent manner (Fig. 4). The fermentation broth, diluted 10-fold, reduced fusarium wilt, Rs damping-off, and Pu damping-off by 77.78, 83.33, and 71.67%, respectively. Rs damping-off development was suppressed by culture broth at 10-fold dilution to a similar extent as that of the commercial fungicide Jalrokend at 1000-fold dilution. Furthermore, the BuOH extract at 300 μg/ml effectively suppressed fusarium wilt and Rs damping-off by 77.78 and 83.33%, respectively.

## Discussion

Several antagonistic bacteria—including *Bacillus* [37, 38], *Pseudomonas* [39, 40], and *Streptomyces* [41-43] species—have been employed as biocontrol agents against fungal plant diseases. Among these, *Streptomyces* species exhibit favorable traits such as enhanced nutrient uptake, nitrogen fixation [44], siderophore production [45], plant hormone production, plant growth promotion, and antimicrobial metabolite production [46-48]. Owing to their prolific antimicrobial compound biosynthesis, *Streptomyces* have been widely used in agronomic applications [49].

During the screening of > 300 antagonistic actinomycetes isolated from soil, the culture filtrate of JCK-6116 exhibited strong antifungal activity against *Rs* AG-4, *Pc*, and *Foc* with MIC values of 0.16, 1.25, and 2.5%, respectively (Table S6). Analysis of the 16S rRNA gene confirms that the JCK-6116 strain belongs to the genus *Streptomyces*. From the culture filtrate of *Streptomyces* sp. JCK-6116, three different rimocidins—rimocidin A, B, and C—were isolated as antifungal metabolites against phytopathogenic fungi. Rimocidins belong to the polyene macrolide class of compounds produced by *Streptomyces* species [50]. This group includes commercially important antifungal agents such as amphotericin B, pimaricin, and nystatin. Structurally, rimocidins are glycosylated polyketide composed of a macrolactone ring connected to a D-mycosamine moiety (Jeon *et al*., 2016). Rimocidin A (**1**) was first reported as an antifungal antibiotic produced by *S. rimocus* by Davisson J,Tanner F, Jr,Finlay A, Solomons I [23]. Subsequently, rimocidin A and C (**3**; also known as CE-108) were isolated from *S. diastaticus* 108 [25]. Rimocidin A and B (**2**; also known as BU16) were isolated from *S. mauvecolor* BU16 [24]. This finding indicates that strain JCK-6116 can naturally produce three distinct rimocidins.

The biological activities of polyenes have been extensively characterized in previous research. For example, [25] report the antifungal activity of rimocidin A and C (CE-108), isolated from *Streptomyces diastaticus* 108, against multiple fungal pathogens. Rimocidin A demonstrated two times higher activity than that of rimocidin C against *Candida*, *Microsporum*, and *Trichophytum* species. However, both compounds exhibited similar efficacy against *Aspergillus* species and *Fusarium oxysporum*, with a MIC of 8 μg/ml. Subsequently, rimocidin C derivatives were isolated from *S. diastaticus* 108 via gene disruption techniques [51]. In this study, rimocidin A exhibited > 18 times higher antifungal activity than that of rimocidin C against *Issatchenkia orientalis*, *Filobasidiella neoformans*, *Aspergillus niger*, and *Penicillium chrysogenum*. Converting the side chain carboxyl group of rimocidin A and C into an amide group led to enhanced antifungal activity. Subsequently, rimocidin A (MIC = 2 μg/ml) exhibited stronger antifungal activity against *R. solani* than that of rimocidin B (BU16) (MIC = 8 μg/ml) [24]. However, a comparison of antifungal activities of rimocidins A, B, and C has not been studied.

The mechanism of action of antifungal polyenes has been described in previous studies. Amphotericin B—a well-known polyene antifungal—exerts its effect by binding to ergosterol in the bilayer membrane, forming ion channels or creating extramembranous sponge-like aggregates that extract ergosterol from the lipid membrane of yeast cells [19]. Since the primary difference among these three compounds lies in their hydrocarbon side chains, we hypothesize that increased side chain complexity would enhance membrane permeability. This could occur by facilitating the formation of larger ion channels or more effective aggregates. Therefore, rimocidin A—which possesses the most complex side chain—is expected to induce the greatest degree of cellular membrane leakage. Our *in vitro* antifungal activity data also support this hypothesis, showing a clear trend: rimocidin A > rimocidin B > rimocidin C. This trend strongly suggests that increasing the complexity of the hydrocarbon side chain enhances antifungal potency. These findings provide a foundational basis for the rational design and synthesis of more effective polyene-based antifungal compounds. Although ergosterol is the major sterol in fungal cell membranes, the sterol biosynthesis pathway is absent in Oomycetes because of the loss of key enzymes that catalyze this process [52]. Consequently, polyenes could not inhibit Oomycetes owing to the absence of sterol on the bilayer membrane. Our findings also indicate that rimocidin A, B, and C did not inhibit the mycelial growth of *Phytophthora* spp. and *Pythium* spp. (Table 2). However, the JCK-6116 strain exhibited *in vitro* and *in vivo* anti-oomycete activity against *P. ultimum* (Fig. 2 and Table S6). The strain also produced several hydrolytic enzymes, including chitinase, protease, and lipase, among others, but lacked cellulase. These findings suggest that JCK-6116 may also produce anti-oomycete metabolites. Overall, this study indicates that JCK-6116 has a promising potential to be exploited for the control of soil-borne plant diseases caused by fungi and Oomycetes.

In this study, the culture broth of JCK-6116 effectively suppressed fusarium wilt, *Rs*, and *Pu* damping-off diseases on cucumber, demonstrating efficacy comparable to that of the corresponding commercial fungicides. *Foc*, *Rs* AG-4, and *Pu* are major soil-borne plant diseases globally, causing significant economic losses. These diseases cause significant damage to cucumber crops, leading to significant yield losses. To manage them, various control strategies have been employed, including the application of resistant varieties, physical-chemical methods, and biological control, with biocontrol agents emerging as a prominent approach. Biocontrol agents, primarily bacteria and their metabolites offer advantages, including long-lasting efficacy and minimal to no residual toxicity in crops and the environment. Consequently, they are increasingly replacing synthetic fungicides for managing various plant diseases. For example, *Bacillus subtilis* JNF2 demonstrated a biocontrol efficacy of 81.33% against fusarium wilt on cucumber, which is higher than that of commercial hymexazol [53]. *Paenibacillus polymyxa* PJH16, known for its significant plant growth-promoting ability, suppressed fusarium wilt by 94% *in vivo* [54]. Moreover, *S. luteoverticillatus* B4—isolated from the cucumber interrhizosphere—effectively suppressed the foc wilt by 56% in pot experiments [55]. *S. albidoflavus* W68, which produces candicidin isomer, reduced *Rhizoctonia solani* rot on cucumber by 74.62% [56]. Culture broth of *Streptomyces* sp. JCK-6019, at a 10-fold dilution, effectively suppressed fusarium wilt and Rs damping-off with control rates of 62.5% and 100%, respectively. In another study, the disease severity of *Pythium aphanidermatum* damping-off on watermelon was significantly reduced following seed treatment with *S. murinus* JKTJ-3 [57], which produces actinomycin D and chitinase. *Streptomyces* sp. JCK-6116, producing rimocidins and chitinase, effectively controlled cucumber diseases caused by Foc, Rs, and Pu. The concentration of rimocidin A, B and C in the culture filtrate of JCK-6116 reached 438.87, 141.77, and 88.27 μg/ml, respectively, after 7 days of fermentation in the GSS medium, as determined by standard curves (Fig. S2). The amounts of rimocidin B and C produced were 32.3 and 20.11% of the rimocidin A concentration. In previous sudy, *S. rimosus* M527-S38, a mutant strain that obtained by conjugating of plasmid pAN with the chromosome of *S. rimosus* M527, produced 284.6 mg/l of rimocidin A, representing a 44.1%increase in production compared to the wild type strain strain M527 (197.5 μg/ml) [58]. Furthermore, over-expressing acetyl-CoA carboxylase in strain M527-ACC increased the yield of rimocidin A to 320.7 μg/ml [59]. Our findings show that JCK-6116 naturally produced a higher content of rimocidin A (438.87 μg/ml) than the aforementioned *S. rimosus* mutant strains. Consequently, *Streptomyces* sp. JCK-6116 holds significant potential as a biocontrol agent for managing soil-borne fungal and Oomycetes diseases.

## Conclusion

In this study, *Streptomyces* sp. JCK-6116, isolated from rhizosphere soil, demonstrated potent antifungal and anti-oomycetes activities against 20 fungal and Oomycetes species in dual culture assay, respectively. The strain produced three rimocidin variants, which exhibited broad-spectrum antifungal efficacy against fungi but showed no inhibitory effect on Oomycetes. Among the three compounds, rimocidin A exhibited the most potent antifungal activity. The culture broth of JCK-6116 demonstrated high efficacy in suppressing fusarium wilt and damping-off diseases on cucumber caused by *Foc*, *Rs*, and *Pu*. In contrast, its BuOH extract exhibited high control efficacy against the two fungal diseases but not against Oomycetes disease. These findings suggest that JCK-6116 may also produce anti-oomycete metabolites, warranting further research to identify the active compounds. Overall, this study highlights the high potential of JCK-6116 as a microbial fungicide for managing various soil-borne diseases caused by fungi and Oomycetes.

## Supplemental Materials

Supplementary data for this paper are available on-line only at http://jmb.or.kr.



## Figures and Tables

**Table 1:** NMR spectroscopic data for compounds 1, 2, and 3 purified from *Streptomyces* sp. JCK-6116.

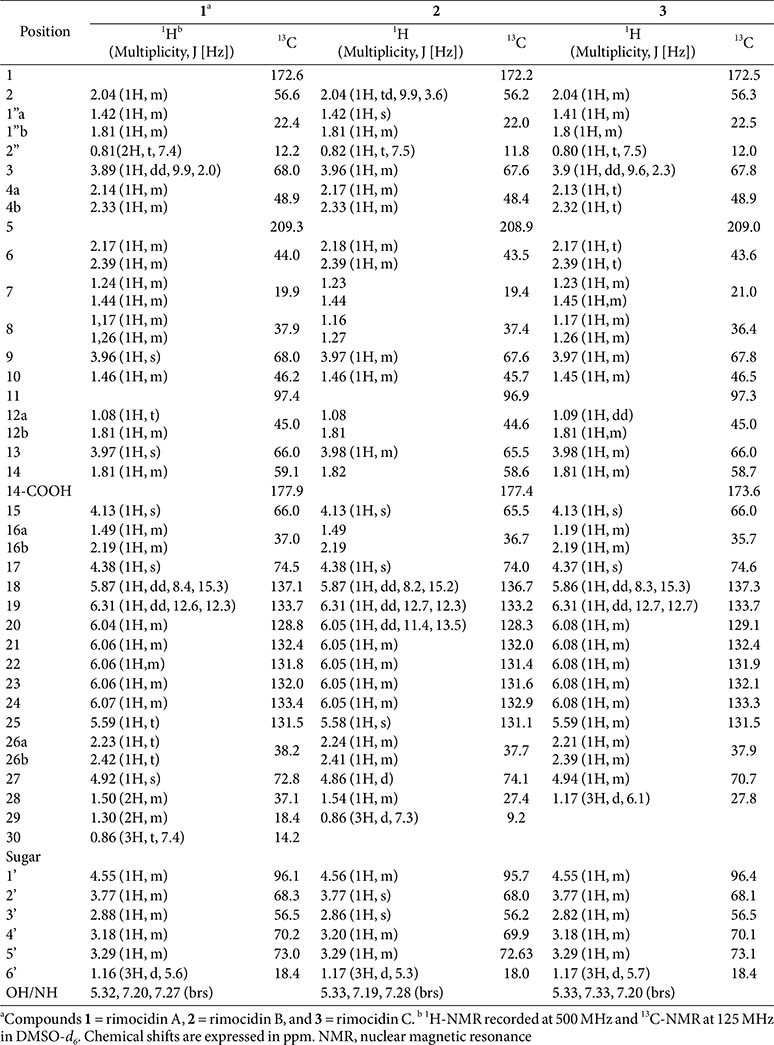

**Table 2:** *In vitro* antifungal activity of rimocidins A, B, and C isolated from *Streptomyces* sp. JCK-6116.