# Supplementary material for: Harnessing Rimocidins-Producing Streptomyces sp. JCK-6116 as a Sustainable Fungicide for Biocontrol of Cucumber Soil-Borne Diseases
Source: J Microbiol Biotechnol. 2025 Oct 28;35:e2508023. doi: 10.4014/jmb.2508.08023 (PMC12603372; doi:10.4014/jmb.2508.08023)
Supplement: Supplementary file 1 [file jmb-35-e2508023-supple.pdf]

1.1 J. Microbiol. Biotechnol. 2025. 35: e2508023

https://doi.org/10.4014/jmb.2508.08023

Supplementary Figures and Tables

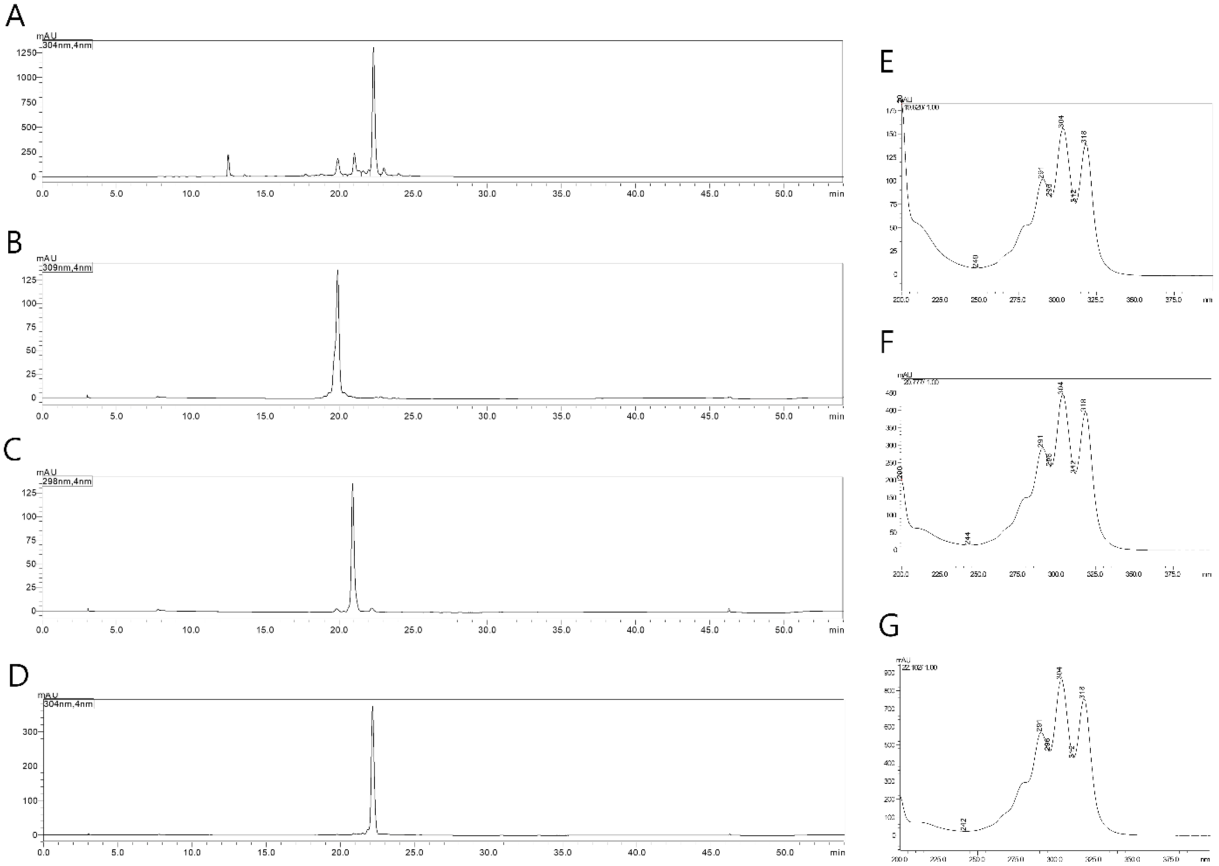

Fig. S1. HPLC chromatograms of BuOH extract (A) rimocidin C **(B)** rimocidin B **(C)** rimocidin A **(D)** and UV spectra of rimocidin C **(E)** rimocidin B **(F)** rimocidin A **(G)** which derived from JCK-6116

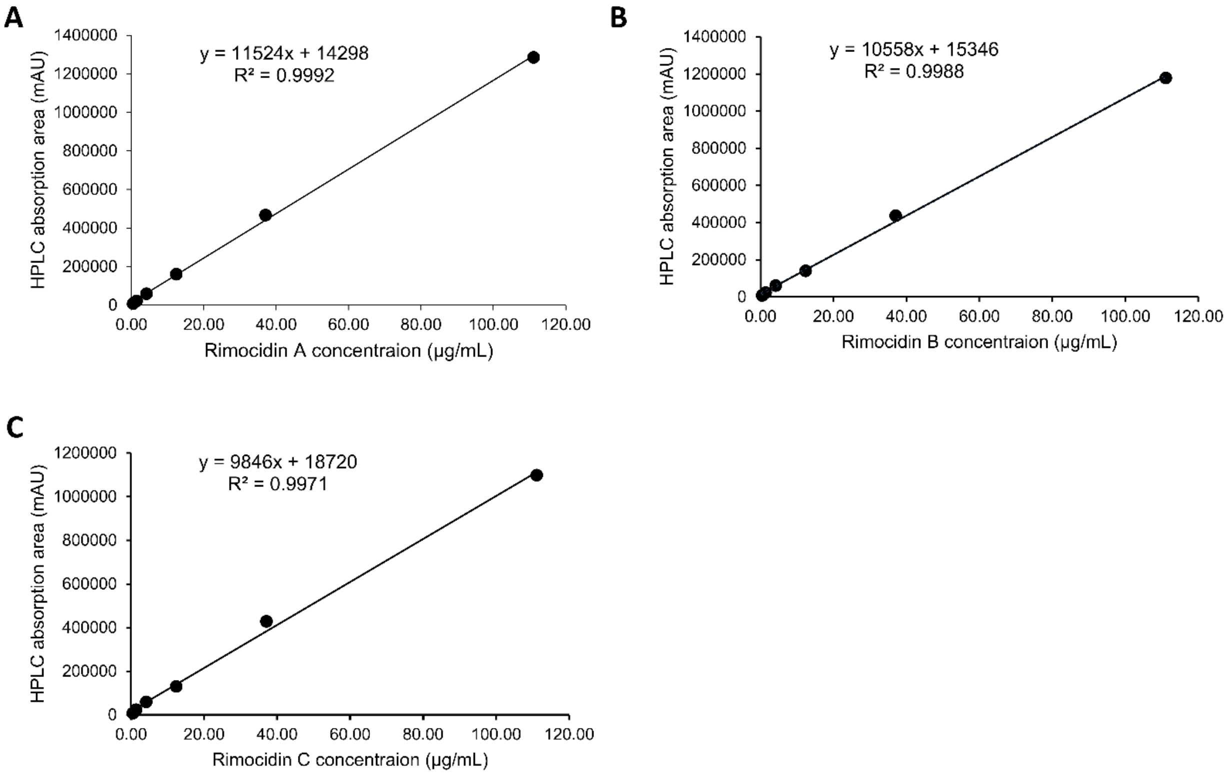

Fig. S2. Standard curve of rimocidins A, B, and C.

Table S1. List of fungi and oomycetes used in this study.

Test fungus	Disease	Host and/or origin[*]
Armillaria rolfsii KACC40170	Southern blight	Apple, KACC
Botryosphaeria dothidea KACC45481	Apple botryosphaeria canker	Crab apple, KACC
Botrytis cinerea KACC40574	Grey mold disease	Tomato, KACC
Colletotrichum coccodes KACC40010	Anthracnose	Red pepper, KACC
Collectotrichum horri	Anthracnose	Persimmon, KRICT
Curvularia lunata KACC41020	Curvularia blight	Turf grass, KACC
Cryphonectria parasitica KACC40323	Chestnut blight	Chestnut, KACC
Fusarium fujikuroi	Bakana	Rice, KRICT
Fusarium graminearum Z-3639	Fusarium head blight	Wheat (Nguyen et al., 2021)
Fusarium oxysporum f.sp. *lycorpersici* KR5	Fusarium wilt	Tomato, KRICT
Fusarium oxysporum f.sp. *niveum*	Fusarium wilt	Watermelon, KRICT
Fusarium oxysporum f.sp. *raphanin*	Fusarium wilt	Radish, KRICT
Fusarium verticillioides	Ear rots	Maize, KRICT
Gaeumannomyces graminis	Take-all	Turfgrass, DJ
Magnaporthe oryzae KACC46522	Rice blast	Rice, KACC
Magnaporthiopsis poae	Summer patch	Kentucky blue grass, DJ

Ophiostoma ulmi KACC40252	Dutch elm disease	Elm, KACC
Pseudocercospora circumcissa	Cherry leaf spot	Cherry tree, KRICT
Raffaelea quercus-mongolicae KACC44403	Oak wilt	Oak, KACC
Rhizoctonia solani AG-4 KACC40140	Damping off disease	Cucumber, KACC
Rhizoctonia solani	Brown patch disease	Zoysia grass, DJ
Rhizoctonia cerealis	Sharp eyespot	Wheat, KRICT
Clarireedia jacksonii	Dollar spot disease	Creeping bentgrass, DJ
Valsa ceratosperma KACC40830	Canker	Apple, KACC
Phytophthora cactorum KACC40166	Phytophthora stem rot	Apple, KACC
Phytophthora cambivora KACC40159	Phytophthora collar rot	Apple, KACC
Phytophthora capsici KACC40158	Phytophthora blight	Pepper, KACC
Phytophthora cinnamomi KACC40182	Mating type, root rot, blight	Larch, KACC
Pythium ultimum KACC40705	Damping off	Cucumber, KACC

*KACC = Korean Agriculture collection culture, KRICT = Korean Research Institute of Chemical Technology, DJ=Daejeong-Golf Engineering Co. Ltd.

Table S2. Cultural characteristics of *Streptomyces* sp. JCK-6116.

JCK-6116	Cultural characteritistics		
	Aerial mycelium	Substrate mycelium	Soluble pigment
ISP1	White	Dark-brown	Brown
ISP2	White	Dark-grey	brown
ISP3	White-violet	Pale-yellow	None
ISP4	White-pink	Pale-yellow	None
ISP5	White	White	None
Bennet's	White	Brown	None
PDA	white	Brown	None

Table 3. Carbon utilization of JCK-6116.

No.	Carbohydrate sources	JCK-6116	No.	Carbohydrate sources	JCK-6116
1	Glycerol	-	26	Salicin	-
2	Erytrol	-	27	D-Celobiose	-
3	D-Arabinose	-	28	D-Maltose	-
4	L-Arabinose	-	29	D-Lactose	-
5	Ribose	-	30	D-Melibiose	-
6	D-Xylose	-	31	D-Sucrose	+
7	L-Xylose	-	32	D-Trehalose	-
8	D-Adonitol	-	33	Inuline	-
9	Metil-β-D-Xylopyranoside	-	34	D-Melezitose	-
10	Galactose	-	35	D-Rafinose	-
11	Glucose	-	36	Starch	-
12	Fructose	-	37	Glycogen	-
13	Manose	-	38	Xylitol	-
14	Sorbose	-	39	Gentiobiose	-
15	Rhamnose	-	40	D-Turanose	-
16	Dulcitol	-	41	D-Lyxose	-
17	Inozitol	-	42	D-Tagatose	-
18	Manitol	-	43	D-Fucose	-
19	Sorbitol	-	44	L-Fucose	-
20	Metil-α-DManopyranoside	-	45	D -Arabitol	-
21	Metil-α-DGlucopyranoside	-	46	L -Arabitol	-
22	N-Acetyl-glucosamine	-	47	Potasium gluconate	-
23	Amygdaline	-	48	2 -Ketogluconate	-
24	Arbutine	-	49	5 -Ketogluconate	-
25	Esculin	+			

+ Positive; - negative

Table S4. Enzyme activity of strain JCK-6116.

No.	Enzyme assayed for	JCK-6116
1	Control	-
2	Alkaline phosphatase	+
3	Esterase (C4)	+
4	Esterase Lipase (C8)	+
5	Lipase (C14)	+
6	Leucine arylamidase	+
7	Valinearyl lamidase	+
8	Cysteine arylamidase	+
9	Trypsin	+
10	α-Chymotrypsin	+
11	Acid phosphatase	+
12	Naphthol-AS-BI-phosphohydrolase	+
13	α-Galactosidase	-
14	β-Galactosidase	-
15	β-Glucuronidase	-
16	α-Glucosidase	-
17	β-Glucosidase	+
18	N-acetyl-β-gucosaminidase	+
19	α-Mannosidase	+
20	α-Fucosidase	-
21	Protease	+
22	Chitinase	+
23	Cellulase	-

+ Positive; - negative

Table S5. Carbon utilization of strain JCK-6116 and *Streptomyces mauvecolor* ATCC 29835.

Characteristics		JCK-6116	*Streptomyces mauvecolor* ATCC 29835
Carbon utilization	D-glucose	-	+
	Arabinose	-	+
	Sucrose	+	-

+ Positive; - negative

Table S6. *In vitro* antifungal activity of culture filtrate against mycelial growth of fungi and oomycete.

No.	Number of Isolates	MIC (%)					
		Pythium ultimum	*Rhizoctonia solani* AG-4	*F.oxy.* f.sp. *cucumberinum* KR5	*F graminearum*	*S. homoeocarpa*	*R. solani*
1	JCK-6016	10	>10	-	1.25	>10	-
2	JCK-6022	5	5	5	1.25	10	-
3	JCK-6030	1.25	0.63	2.5	>10	-	-
4	JCK-6032	>10	0.16	2.5	>10	5	-
5	JCK-6036	1.25	10	>10	2.5	5	-
6	JCK-6043	0.63	>10	>10	1.25	2.5	-
7	JCK-6063	0.16	1.25	1.25	2.5	10	2.5
8	JCK-6068	1.25	1.25	5	2.5	0.63	1.25
9	JCK-6072	0.63	0.16	-	1.25	1.25	2.5
10	JCK-6095	10	>10	-	>10	5	-
11	JCK-6099	10	0.63	5	>10	2.5	10
12	JCK-6102	1.25	0.16	2.5	2.5	0.63	1.25
13	JCK-6110	1.25	1.25	1.25	2.5	2.5	0.625

14	JCK-6116	1.25	0.16	2.5	2.5	0.63	0.31
15	*Streptomyces* sp. JCK-6141 (Nguyen et al., 2021)	0.31	0.63	0.31	0.08	0.63	2.5

- -: No activity was detected at test concentration
